# Comparison of the effects of air-powder abrasion, chemical decontamination, or their combination in open-flap surface decontamination of implants failed for peri-implantitis: an ex vivo study

**DOI:** 10.1007/s00784-020-03578-w

**Published:** 2020-09-25

**Authors:** Nicola Pranno, Maria Paola Cristalli, Fabio Mengoni, Ilaria Sauzullo, Susanna Annibali, Antonella Polimeni, Gerardo La Monaca

**Affiliations:** 1 Department of Oral and Maxillofacial Sciences, Sapienza University of Rome, Rome, Italy; 2 Department of Biotechnologies and Medical Surgical Sciences, Sapienza University of Rome, 6. Caserta St., 00161 Rome, Italy; 3 Department of Public Health and Infectious Diseases, Sapienza University of Rome, Rome, Italy

**Keywords:** Air-powder abrasion, Chemical decontamination, Peri-implantitis

## Abstract

**Objectives:**

To compare, using an ex vivo model, the biofilm removal of three surface decontamination methods following surgical exposure of implants failed for severe peri-implantitis.

**Materials and methods:**

The study design was a single-blind, randomized, controlled, ex vivo investigation with intra-subject control. Study participants were 20 consecutive patients with at least 4 hopeless implants, in function for >12 months and with progressive bone loss exceeding 50%, which had to be explanted. Implants of each patient were randomly assigned to the untreated control group or one of the three decontamination procedures: mechanical debridement with air-powder abrasion, chemical decontamination with hydrogen peroxide and chlorhexidine gluconate, or combined mechanical-chemical decontamination. Following surgical exposure, implants selected as control were retrieved, and afterwards, test implants were decontaminated according to allocation and carefully explanted with a removal kit. Microbiological analysis was expressed in colony-forming-units (CFU/ml).

**Results:**

A statistically significant difference (*p* < 0.001) in the concentrations of CFU/ml was found between implants treated with mechanical debridement (531.58 ± 372.07) or combined mechanical-chemical decontamination (954.05 ± 2219.31) and implants untreated (37,800.00 ± 46,837.05) or treated with chemical decontamination alone (29,650.00 ± 42,596.20). No statistically significant difference (p = 1.000) was found between mechanical debridement used alone or supplemented with chemical decontamination. Microbiological analyses identified 21 microbial species, without significant differences between control and treatment groups.

**Conclusions:**

Bacterial biofilm removal from infected implant surfaces was significantly superior for mechanical debridement than chemical decontamination.

**Clinical relevance:**

The present is the only ex vivo study based on decontamination methods for removing actual and mature biofilm from infected implant surfaces in patients with peri-implantitis.

## Introduction

Implant therapy is an effective and predictable method to replace missing teeth with high long-term success and survival rates. Nevertheless, biological complications, i.e., peri-implant mucositis and peri-implantitis, due to the local inflammatory reaction of marginal soft tissues to the biofilm may happen.

Peri-implantitis is an increasing problem, with a wide range of the prevalence, ranging between 9.25 and 12.8% at the implant level, and between 17 and 22% at the patient level, due to differences in clinical case definitions [1–3].

Peri-implantitis is “a plaque-associated pathological condition, characterized by inflammation in the peri-implant mucosa and subsequent progressive loss of supporting bone” [4].

As peri-implantitis is the effect of an infection process due to the formation of bacterial biofilm on implant surfaces, the target of treatments, either non-surgical or surgical, is to control bacterial infection and peri-implant inflammation. The goal is to stop the disease progression, which can gradually lead to implant loss, and to preserve healthy tissues around functioning implants.

Different strategies in implant surface decontamination, such as mechanical, chemical, photodynamic, and laser therapies, either alone or in various combinations, have been suggested during peri-implant surgery for reducing the bacterial load and removing the biofilm [5, 6].

In mechanical debridement, ultrasonic scaler’s specific tips, curettes of different materials (stainless steel, titanium, coated carbon fiber, Teflon, or plastic), powdered air-abrasive systems, rubber cups, titanium brushes, and abrasive pumice have been used to clean previously contaminated implant surfaces [5–8].

Chemical decontamination consists in topical applications on implant surfaces with saline solution, delmopinol, chlorhexidine, cetylpyridinium chloride (CPC), tetracycline, minocycline, doxycycline, citric acid at pH 1, hydrogen peroxide, EDTA, or 35% phosphoric acid gel [5, 6].

Photodynamic therapy (PDT) makes use of the diode laser irradiation and photosensitizer solution combination [5, 6, 9]. For laser decontamination, different types of devices, such as Erbium, Chromium: Yttrium, Scandium, Gallium, Garnet (Er,Cr:YSGG), Neodymium-doped Yttrium Aluminum Garnet (Nd:YAG), Erbium: Yttrium Aluminum Garnet ( Er:YAG), Gallium, Aluminum Arsenide ( GaAlAs), carbon dioxide (CO2), are used [5, 6].

However, none of these modalities has shown effectiveness in recovering peri-implant health and there is no consensus on the best available treatment to provide satisfactory implant surface decontamination [5, 6].

The present study aimed to compare, using an ex vivo model, the efficacy of mechanical debridement with sodium bicarbonate and glycine powders, chemical decontamination with hydrogen peroxide and chlorhexidine gluconate, and combined mechanical-chemical decontamination in the biofilm removal following surgical exposure of implants failed for severe peri-implantitis.

## Materials and methods

### Study design

The study was designed as a single-blind, randomized, controlled, ex vivo investigation with intra-subject control to compare the semiquantitative concentrations of colony-forming units (CFU/ml) and to assess the qualitative microbial composition on the surface of retrieved infected implants clinically treated with three different decontamination methods.

### Study population

Study participants were recruited from patients referred for treatment of peri-implantitis to the Oral Surgery Unit, Policlinico Umberto I, “Sapienza” University of Rome, Italy, between February 2018 and October 2019.

To be included, the patients had to meet the following criteria: (1) at least four osseointegrated implants (unit of statistical analysis) without any restriction about brands, types, and surface, functioning for > 12 months; (2) progressive bone loss exceeding 50% of the implant length detected on standard intraoral radiographs; (3) presence of bleeding on gentle probing and/or suppuration. In patients with more than 4 hopeless implants, only 4 with the most severe defect were involved in the study.

The following exclusion criteria were applied: implant mobility, mechanical debridement carried out in the previous 3 months, any peri-implant treatment in the past 6 months, antibiotic therapy during 10 days before surgery.

Each patient received detailed descriptions of the procedure, after which written informed consent was obtained. The protocol was in accordance with the 1975 Declaration of Helsinki on medical protocols and ethics and its later amendments. The study protocol was approved by the Department of Oral and Maxillofacial Sciences-Sapienza, University of Rome, Italy (Protocol identifying number: 0001558).

### Randomization

Before the start of the study, selected implants of each patient were randomly assigned to one of the four groups (untreated control, mechanical debridement, chemical decontamination, mechanical debridement combined with chemical decontamination) using a list of random numbers generated using CLINSTAT software (Martin Bland, York, UK) and sequentially numbered opaque sealed envelopes. The envelopes were opened by the surgeon, and the assigned decontamination method was carried out. The microbiologist assessor was unaware of the delivered treatment. The treatment code was not revealed until all microbiological tests had been completed, and the data file had been established.

### Decontamination procedures

In all groups, treatment was performed by the same surgeon (G.L.M), experienced in the protocol of surface decontamination and reconstructive surgery of peri-implantitis defects [10].

Immediately prior to intervention, patients rinsed with a 0.2% chlorhexidine digluconate solution (Corsodyl, GlaxoSmithKline Consumer Healthcare S.p.A. Baranzate, Milan, Italy) for 2 min. Under local anesthesia with 2% mepivacaine and 1:100,000 adrenalin (Carbocaine, AstraZeneca, Milan, Italy), the prosthetic supra-structure was removed and mucoperiosteal flaps were raised at the buccal and oral aspects. Granulation tissue was removed with titanium curettes (Hufriedy, Chicago, IL, USA) to expose the implant threads and bone defect. Supra- and intrabony implant surfaces were thoroughly irrigated with sterile saline solution for 1 min.

Before decontamination procedures, the implant selected as control was retrieved. Then, each test implant was singularly treated according to allocation and explanted. In order not to influence the results of the other groups, adjacent implants were carefully coated with gauzes soaked with sterile saline solution or, in some cases, postponing overlying soft tissues incision. All implants were explanted using the Implant Retrieval Kit–Nobel Biocare. A specific retrieval instrument mounted through an adapter to the manual torque wrench was put into the implant, which was pulled out with anti-rotational movements and directly transferred in the single tubes, to avoid contamination by bacteria of the oral cavity [11].

At the end of retrieval procedures, mucoperiosteal flaps were repositioned and stabilized with resorbable interrupted sutures (5-0 Vicryl, Ethicon S. p. A. Johnson & Johnson, Pratica di Mare, Rome, Italy), which were removed after 2 weeks. The postoperative antibiotic protocol included amoxicillin (875 mg) plus clavulanic acid (125 mg) (Augmentin, GlaxoSmithKline S.p.A., Verona, Italy) twice daily, and metronidazole (250 mg) (Flagyl, Zambon, Milan, Italy) three times daily for 7 days. Analgesia was achieved with ketoprofen (Ibifen, Istituto Biochimico Italiano G. Lorenzini S. p. A., Aprilia, Latina, Italy) 200 mg for a maximum of three times daily according to individual needs.

### Decontamination modalities

**Fig. 1:**
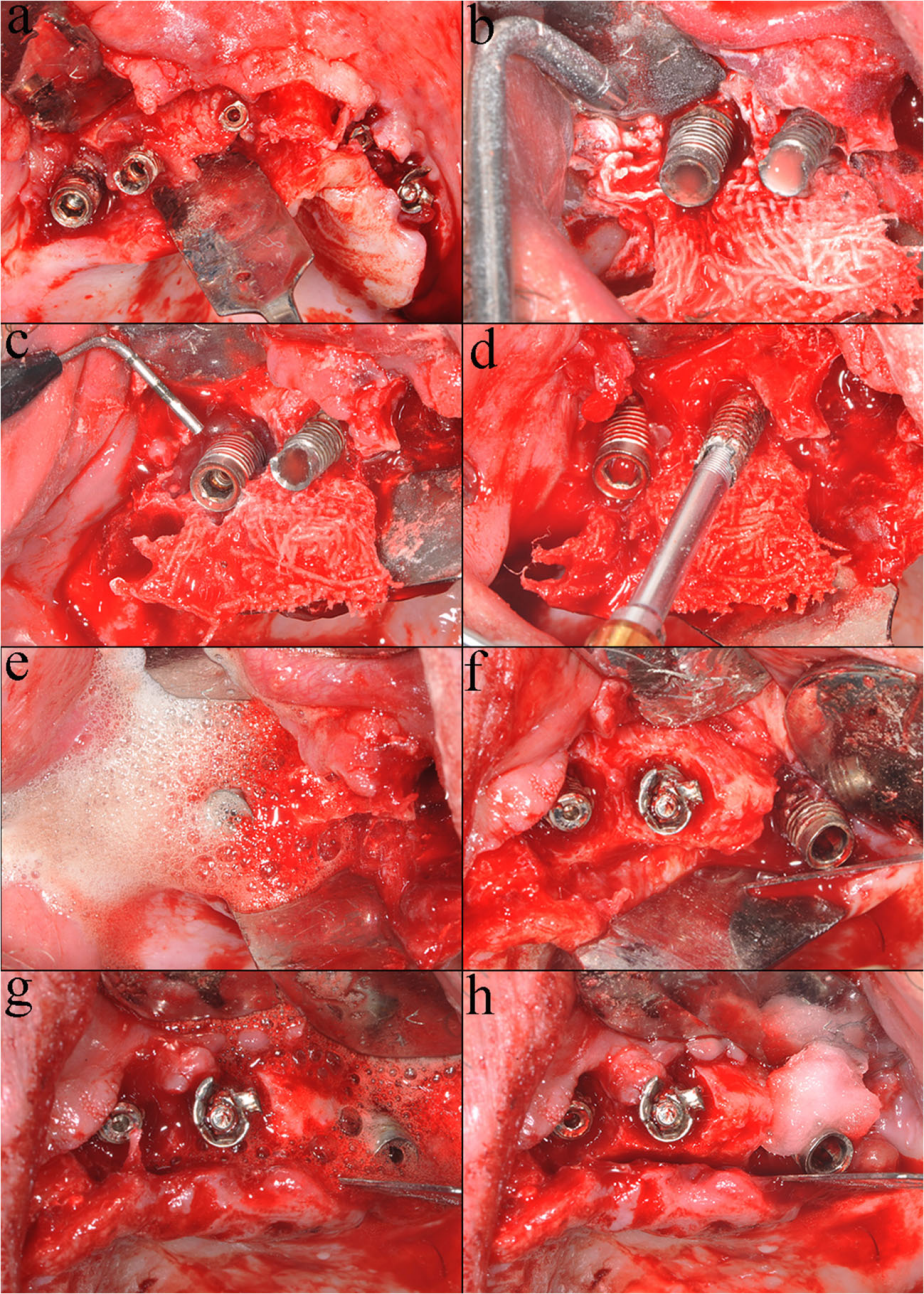
Implant surface decontamination: **a** the mucoperiosteal flap raised; **b** sodium bicarbonate powder-based air abrasion; **c** glycine powder-based air abrasion; **d** retrieval of the mechanically decontaminated test implant; **e** chemical decontamination of the remaining implant performed after mechanical debridement; **f** implants before chemical decontamination; **g** hydrogen peroxide at 3%; **h** chlorhexidine gluconate at 2%

Mechanical debridement was performed with sodium bicarbonate and glycine powders in sequence using the same powered air-abrasion device (PROPHYflex™ 3 with perio-tip, KaVo, Biberach, Germany) (Fig. 1b, c). The working distance and angulation were individually selected according to the bone defect, and the instrumentation time was for 2 min per implant [12, 13]. Chemical decontamination was carried out with cotton pellets soaked with hydrogen peroxide at 3% for 2 min, followed by 0.2% chlorhexidine for an additional 1 min (Fig. 1f, g, h). Mechanical debridement supplemented by chemical decontamination was performed in the same ways above mentioned (Fig. 1b, c, e).

### Microbiological sampling and analysis

Each retrieved implant, labeled with a code number, was immediately transferred to the microbiology laboratory into a single tube containing thioglycollate medium and processed via sonication within 6 h after its removal.

Tubes were vortexed for 30 s using vortex mixer (VELP Scientifica), sonicated at a frequency of 40 kHz at 22 °C for 5–7 min (BANDELIN Electronic GmbH & Co. KG, Berlin, Germany), and then vortexed again for 30 s to obtain a biofilm disintegration. Sonication fluid was centrifuged (3200 rpm for 15 min), the supernatant was aspirated, and the sediment was resuspended in 100 μl of medium. A volume of 10 μl of medium was placed onto aerobic Columbia sheep blood agar plates and onto anaerobic Schaedler sheep blood agar and incubated for 5 and 10 days, respectively. The semiquantitative estimate was made by counting on plates and expressed in colony-forming units (CFU/ml). The minimum detection level was 5 CFU/ml. Microbial identification was performed by Bruker MALDI-TOF MS (Bruker Daltonics, Billerica, MA, USA). Species found on plates were identified for each implant.

### Sample size calculation

The effect size value was calculated based on the mean concentrations of CFU/ml for each group evaluated in the first five patients (16 implants), using statistics software (GPower 3.1.9.2, Heinrich-Heine-Universität, Düsseldorf, Germany). A power analysis using the repeated measures ANOVA with four measurements, an alpha level of 0.05, and a medium effect size (f = 0.57) showed that 80 implants would be adequate to obtain 95% power in detecting a statistical difference in the CFU/ml between control and treatment groups assuming a loss of 20% of the sample during all procedures [14].

### Statistical analysis

The implant was chosen as the unit for the statistical analysis.

Data were evaluated using standard statistical analysis software (version 20.0, Statistical Package for the Social Sciences, IBM Corporation, Armonk, NY, USA). A database was created using Excel (Microsoft, Redmond, WA, USA). Descriptive statistics including mean ± SD values and percentage were calculated for each variable: concentrations of CFU/ml and microbiological differences.

The Shapiro-Wilk test was used to determine whether or not the data conformed to a normal distribution. As a non-parametric distribution of the concentrations of CFU/ml between four groups was found, a Kruskal-Wallis test was conducted to determine differences in the implant surface detoxification treatments. Pairwise comparisons were performed using Dunn’s procedure [15] with a Bonferroni correction for multiple comparisons. Adjusted *p* values are presented. Fisher’s exact test was used to evaluate the presence of qualitative microbiological difference. In each test, the cut-off for statistical significance was *p* ≤ 0.05.

## Results

Twenty consecutive patients (11 males and 9 females; age 65.75 ± 9.67 years) aged > 18 years were selected for a total of 80 implants with different rough surfaces (4 for each subject). No test or control implants were lost during decontamination procedures or the incubation period. In all groups, no intraoperative complications occurred (e.g., emphysema formation) and the postoperative wound healing was uneventful.

### Semiquantitative microbiological analysis

The semiquantitative microbiological analysis found mean values of 37,800.00 ± 46,837.05 CFU/ml for untreated implants, 531.58 ± 372.07 CFU/ml for implants treated with mechanical debridement, 29,650.00 ± 42,596.20 for implants treated with chemical decontamination, and 954.05 ± 2219.31 for implants treated with combined mechanical-chemical decontamination.

**Fig. 2:**
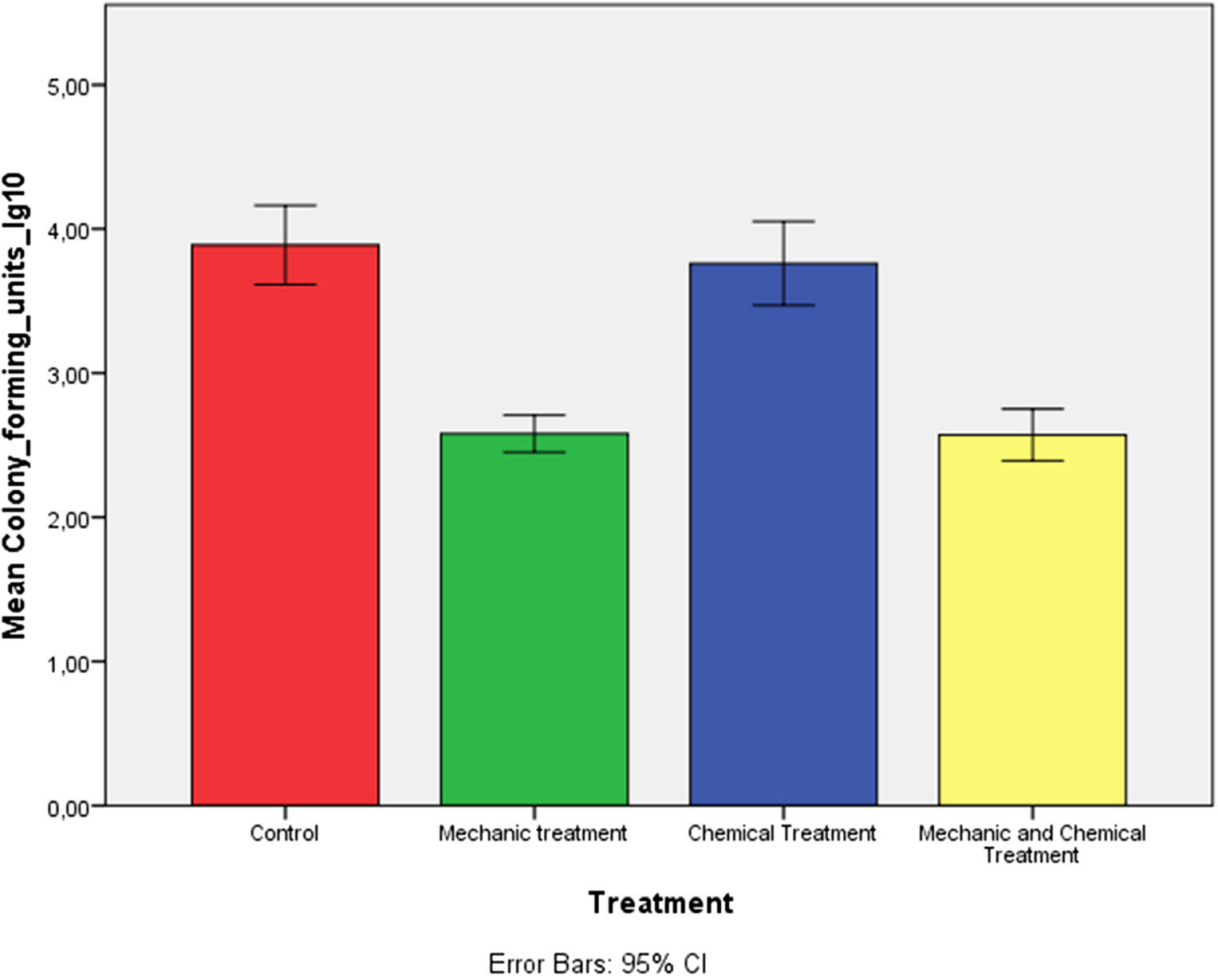
Bar chart based on the log10 of mean CFU. The error bar represented the standard deviation of mean CFU/ml expressed using logarithmic notation. The graph showed a major number of CFU in the control group and in the group treated with chemical agents compared with both groups treated with mechanical debridement and with mechanical debridement combined with chemical decontamination.

The concentrations of CFU/ml for each group were presented in a bar chart illustrating a lower value in mean counts only in the group treated with mechanical debridement and with mechanical debridement combined with chemical decontamination (Fig. 2).

**Fig. 3:**
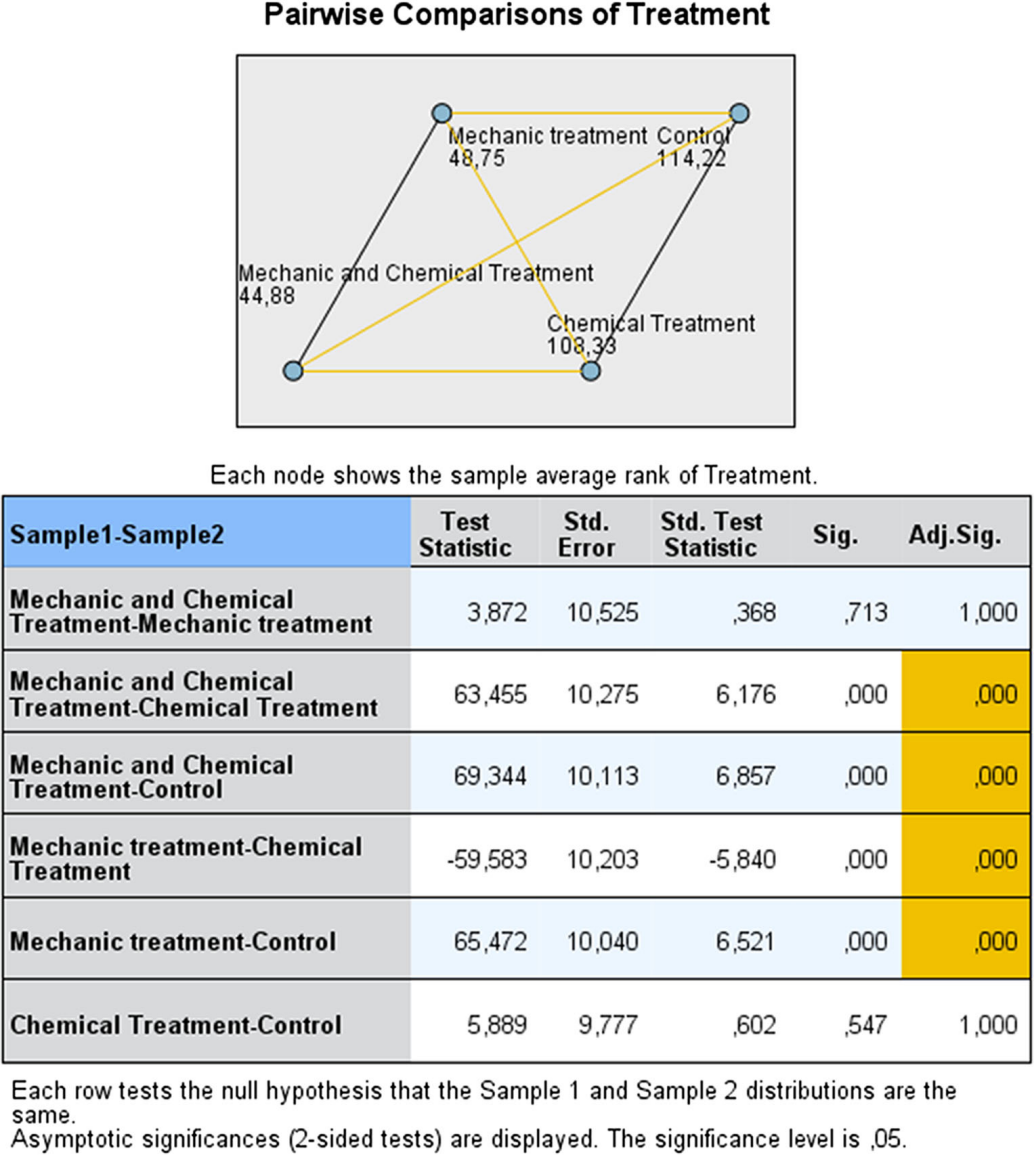
Pairwise comparison of decontamination methods. In the diagram, the numbers and the joining lines reflected respectively the average rank and the pairwise comparison for each group. Orange and black lines reflected pairwise comparisons, respectively statistically significant and not significant. The table below the diagram showed pairwise comparisons in more detail: the first column indicated which pairwise comparison was made and in what direction; the second column reported the test statistic (difference between mean ranks of the two groups); the third and fourth columns presented the standard error and the standardized statistic test; the fifth and the sixth columns showed the unadjusted and adjusted *p* value.

Differences in the concentrations of CFU/ml were statistically significant (*p* < 0.001) when mechanical debridement alone or supplemented by chemical decontamination was compared with implants untreated (positive control group) or treated with chemical decontamination (Fig. 3). Conversely, no statistically significant difference (*p* = 1.000) was found comparing chemical decontamination to untreated implants and mechanical debridement to combined mechanical-chemical decontamination (Fig. 3).

### Microbiological findings

The microbiological analysis of 80 implants affected by peri-implantitis identified 21 microbial species. The most frequent bacteria were *Staphylococcus aureus* (40 implants), *Streptococcus mitis/oralis* (32 implants), and *Staphylococcus epidermidis* and *Streptococcus salivarius* (20 implants). *Enterococcus faecalis* and *Candida albicans* were found in 16 and 12 implants, respectively, and *Pseudomonas aeruginosa* and *Neisseria flavescens* in 8 implants. The frequency of microorganisms detected in control and test implants is reported in Table 1. There was no statistically significant difference in the microbial species between control and treatment groups as assessed by Fisher’s exact test (*p* = 0.917). In short, implants affected by peri-implantitis, regardless of their site in the oral cavity, showed the same microbiota.

**Table 1:** Frequency of microorganisms detected in control and test implants

Types of bacteria	Frequency (implants)	Percent	Valid percent	Cumulative percent
*Micrococcus luteus*	8	3.0	3.0	3.0
*Strep. constellatus*	8	3.0	3.0	6.1
*Strep. mitis/oralis*	32	12.1	12.1	18.2
*Candida albicans*	12	4.5	4.5	22.7
*Strep. salivarius*	20	7.6	7.6	30.3
*Neisseria subflave*	12	4.5	4.5	34.8
*Staf. epidermidis*	24	9.1	9.1	43.9
*Enterococcus faecalis*	16	6.1	6.1	50.0
*Staf. aureus*	40	15.2	15.2	65.2
*Pseudomonas aeruginosa*	8	3.0	3.0	68.2
*Neisseria flavescens*	8	3.0	3.0	71.2
*Strep. parasanguinis*	20	7.6	7.6	78.8
*Strep. pneumoniae*	8	3.0	3.0	81.8
*Strep. vestibularis*	8	3.0	3.0	84.8
*Strep. epidermidis*	8	3.0	3.0	87.9
*Strep. viridans*	8	3.0	3.0	90.9
*Klebsiella oxytoca*	4	1.5	1.5	92.4
*Eikella corrodens*	4	1.5	1.5	93.9
*Lactobacillus paracasei*	8	3.0	3.0	97.0
*Lactobacillus rhamnosus*	4	1.5	1.5	98.5
*Veillonella parvula*	4	1.5	1.5	100.0
Total	264	100.0	100.0	

## Discussion

The results of the microbiologic analysis revealed that implants treated during open-flap surgery with mechanical debridement or with mechanical debridement combined with chemical decontamination presented a statistically significant difference in the concentrations of CFU/ml when comparing to implants untreated (positive control group) or treated with chemical decontamination alone.

A direct comparison of these findings to those previously reported in the literature is difficult because to the best of the authors’ knowledge, no ex vivo studies comparing the efficacy of decontamination methods on the removal of microorganisms from implants surfaces in peri-implantitis defects were available.

In the present study, the choice of sodium bicarbonate and amino-acid glycine air powders for mechanical debridement was due to effectiveness in cleaning contaminated implant surfaces, mainly in the deepest part of peri-implant defects, which is difficult to achieve [16–21]. Air powder abrasive treatments have proven effective in removing biofilm (range 85–100%), and in improving marginal bleeding, bleeding on probing, suppuration, and probing depth in open surgical procedures [7, 22–25]. In in vitro studies, sodium bicarbonate showed to remove more than 84% of bacteria or bacterial products, even if it produced slight to medium surface changes, such as small crater-like defects, rounding, or removal of sharp edges [7]. Amino-acid glycine was also able to remove single bacterial species and plaque from smooth and structured titanium surfaces, and it was less abrasive than sodium bicarbonate powder [7, 12, 26, 27]. The lower momentum and less energy at impact onto implant surface were due to the lower density and hardness and the smaller size of the particles [18]. Sodium bicarbonate larger-sized particles proved to have mechanical removal and impact on implant roughness more significant than glycine small-sized particles [18, 28]. Furthermore, also the greater efficacy in restoring biocompatibility of sodium bicarbonate compared with glycine was probably due to the higher ablation power of its harder and larger particles in the removal of the carbon layer produced by the activity of the biofilm [24].

The different ability of the two powder formulations in removing a biofilm was the rationale at the basis of the sequential use of sodium bicarbonate and glycine [18, 28]. Using small after larger powder particles should be helpful to reach the more difficult areas to clean in the rough implants and to increase surface abrasion because the higher solubility potential reduces the presence of undissolved particles in the water-air stream [28].

Chemical decontamination with hydrogen peroxide plus chlorhexidine gluconate was adopted as these are the most common and extensively antiseptic agents used in periodontics. Furthermore, their association should enhance bactericidal effects, exploiting the synergy between the two different mechanisms of action: the oxidizing effect of hydrogen peroxide and the disruption of the bacterial cell membrane of chlorhexidine gluconate [10].

The limited efficacy of chemical decontamination on infected implant surfaces found in the present investigation was confirmed by the results of other studies [29–31].

Reduced efficacy of the detoxification with hydrogen peroxide 3% was reported in in vitro studies. Zablotsky et al. [32] showed that burnishing with cotton pellets soaked by hydrogen peroxide 3% for 1 min removed significantly more endotoxin from grit-blasted titanium alloy strips contaminated with *Escherichia coli* lipopolysaccharide than untreated controls, but not compared with specimens treated with sterile saline alone. Mouhyi et al. [33] found that hydrogen peroxide at low concentrations was not effective in removing contaminants from commercially pure titanium foils placed on dentures in volunteer patients simulating a peri-implantitis situation. Bürgers et al. [34] assessing the effect of various topical antiseptics on plane titanium specimens contaminated by *Staphylococcus epidermidis*, *Candida albicans*, or *Streptococcus sanguinis*, demonstrated that hydrogen peroxide 3% was solely effective against *Candida albicans*.

Limited effects in decontamination of implants surface were also reported for chlorhexidine. In two randomized, double-blind, controlled trials, treatment of implant surfaces with chlorhexidine 0.12% + cetylpyridinium chloride 0.05% or chlorhexidine 2.0% during resective surgery for peri-implantitis led to a significant reduction of anaerobic bacteria compared with placebo, even if the decrease of bacterial load did not turn into better clinical or radiographic treatment outcomes over 12 months [31, 35]. In an in vivo study on the antimicrobial effectiveness of six different antiseptic solutions on machined titanium specimens exposed overnight in the oral cavity of four volunteers, chlorhexidine demonstrated some efficacy in reducing the bacterial load and significant bactericidal effects against adhering bacteria [29]. The explanation of limited effects of chlorhexidine might be researched both in the brief contact time with implants surface and in copious irrigations with sterile saline solution after use. This situation could interfere with the antimicrobial effects of chlorhexidine in peri-implant lesions, diminishing its binding ability with hard and soft tissue due to the slow-release (substantivity property) [31].

In the present study, the adjunct of antiseptics to mechanical debridement, although proposed in the literature for the treatment of peri-implant infection, showed no statistically significant difference in mean counts of CFU compared with mechanical debridement alone. The data was corroborated by the results of Porras et al. [36], who did not find additional improvements in non-surgical therapy of mucositis when mechanical cleansing and oral hygiene instructions were supplemented by the local irrigation with chlorhexidine 0.12% and topical application of a 0.12% chlorhexidine gel. In a prospective randomized controlled clinical trial on the surgical treatment of severe peri-implantitis, the local application of chlorhexidine 0.2% after the removal of the hard deposits with titanium-coated curettes had no overall impact on clinical and radiological outcomes [37].

Microbiota detected in the present investigation was almost the same at both positive controls and treated implants, regardless of the site in the oral cavity, demonstrating that the removal of bacterial biofilm from the infected implant surfaces is quantitative and not qualitative. The highest prevalence of *Staphylococcus aureus* was consistent with microbiological results at implant sites with varying degrees of inflammation reported in other studies [38–41], and was justified by its high affinity for titanium surfaces [42–44]. The other bacteria found in the present investigation were comparable with those reported in some observational studies, in which peri-implantitis was considered a complex and heterogenous infection, more frequently linked with opportunistic pathogens not primarily associated with periodontitis, such as *Staphylococci*, *Pseudomonas aeruginosa*, *Enterics*, and *Candida* species [45–51]. Indeed, most *Staphylococci* can become from commensal bacteria to pathogens in the presence of implanted medical devices [52, 53]. Furthermore, different species of the genus *Staphylococcus* can colonize implant surfaces affected by peri-implantitis with a prevalence higher than periodontal pathogenic bacteria such as *Tannerella forsythia* and *Porphyromonas gingivalis* [38–40, 54]. Unexpectedly, microbiologic assays did not identify several species of common periodontal pathogenic bacteria.

Strengths of the present ex vivo study were to overcome some limitations of in vitro and animal studies as well as in human clinical trials.

In in vitro studies, specimens (titanium discs, sheets, strips and cylinders) and biofilm contaminants (non-mineralized supragingival plaque, single-species biofilm, bacterial products, indelible non-covering ink) could not represent actual clinical situations, due to differences in the macrostructure (threads shape) and qualitative and quantitative composition of plaque of contaminated implants [7, 12, 20, 21, 27, 29]. Additionally, also the use of custom-made defect models with different morphologies mimicking peri-implant defects was unable to simulate clinical settings, in which treatment outcomes were influenced by many factors such as patient’s characteristics or the presence of the suprastructure [7, 8]. Furthermore, cleaning efficacy of decontamination procedures was different in in vivo or in vitro models, as the anatomical limitations of the oral cavity (e.g., the tongue) and the presence of blood and saliva hamper the accessibility to infected implant surfaces.

In animal studies, the main limitation was the difficulty to directly transfer therapeutic effects on peri-implant infection, which were on average better in animal models (mainly monkeys and dogs) than humans, due to the difference in anatomical characteristics and physiological systems between the two species [55, 56].

In human clinical trials, treatment outcomes are significantly influenced by patient’s factors, such as the level of oral hygiene, peri-implant microbiota, prosthetic designs, immunocompetent characteristics, systemic conditions, history of periodontitis, and cigarette smoking [5]. Further confounder factors were the absence of a true control group (untreated patients) for ethical reasons, the allocation of different types of implants in test and control groups, and the use of clinical parameters instead of the quantification of the residual biofilm as treatment outcomes [30, 57].

Strength points of the present study were the use of failed contaminated implants and not patients as statistical analysis unit, decontamination methods applied before the implants retrieval, untreated implants used as positive control tests, the intra-subject evaluation adopted to overcome the bias on treatment effects due to the implant design and surface, and patient’s factors.

Nevertheless, several limitations were present. The main was the wide range of rough surface implant topographies, which may have influenced the microbial adhesion and complex biofilm formation, and the effectiveness of decontamination methods. Further limitations were the lack of investigation on changes of chemical and physical properties in implant surfaces after instrumentations and the semiquantitative analysis of the peri-implantitis microbiota, instead of culture-independent techniques.

Given increasing worldwide use of dental implants and inevitable augmentation of peri-implantitis cases, additional researches on the decontamination/detoxification of infected titanium surfaces are needed to identify an effective treatment.

## Conclusion

Within above-mentioned limits, in the present ex vivo study, the removal of bacterial biofilm from infected implant surfaces was significantly superior for mechanical debridement with sodium bicarbonate and glycine powders than chemical decontamination with hydrogen peroxide and chlorhexidine gluconate. However, these results must be interpreted with caution, as no decontamination procedure has shown to achieve complete elimination of the biofilm.
